# A Nomogram to Predict Prognostic Value of Red Cell Distribution Width in Patients with Esophageal Cancer

**DOI:** 10.1155/2015/854670

**Published:** 2015-10-12

**Authors:** Gui-Ping Chen, Ying Huang, Xun Yang, Ji-Feng Feng

**Affiliations:** ^1^Department of Oncological Surgery, Zhejiang Cancer Hospital, Hangzhou 310022, China; ^2^Department of Nursing, Zhejiang Cancer Hospital, Hangzhou 310022, China; ^3^Department of Thoracic Surgery, Zhejiang Cancer Hospital, Hangzhou 310022, China

## Abstract

*Objectives*. The prognostic value of inflammatory index in esophageal cancer (EC) was not established. In the present study, we initially used a nomogram to predict prognostic value of red cell distribution width (RDW) in patients with esophageal squamous cell carcinoma (ESCC). *Methods*. A total of 277 ESCC patients were included in this retrospective study. Kaplan-Meier method was used to calculate the cancer-specific survival (CSS). A nomogram was established to predict the prognosis for CSS. *Results*. The mean value of RDW was 14.5 ± 2.3%. The patients were then divided into two groups: RDW ≥ 14.5% and RDW < 14.5%. Patients with RDW < 14.5% had a significantly better 5-year CSS than patients with RDW ≥ 14.5% (43.9% versus 23.3%, *P* < 0.001). RDW was an independent prognostic factor in patients with ESCC (*P* = 0.036). A nomogram could be more accurate for CSS. Harrell's c-index for CSS prediction was 0.68. *Conclusion*. RDW was a potential prognostic biomarker in patients with ESCC. The nomogram based on CSS could be used as an accurately prognostic prediction for patients with ESCC.

## 1. Introduction

Esophageal cancer (EC) is the 8th most common cancer and the 4th leading cause of cancer death worldwide [1]. The incidences vary widely in different countries and regions [2]. In China, the most common pathological type is squamous cell carcinoma (SCC), in contrast to the predominance of adenocarcinoma (AC) in the West [3, 4]. Therefore, a study that takes into account the predominance of esophageal squamous cell carcinoma (ESCC) in China is more and more important.

It is widely accepted that systemic inflammatory response (SIR) plays an important role in cancer progression [5, 6]. Red cell distribution width (RDW) is a widely used laboratory parameter for inflammatory disease [7]. Recent studies revealed that RDW is associated with prognosis in several cancers, such as lung cancer [8] and prostate cancer [9]. However, to our knowledge, no studies regarding the prognostic value of RDW in EC are available.

Therefore, the aim of the current study was to investigate the prognostic role of RDW in patients with ESCC. In addition, we attempt to establish a predictive nomogram to predict the survival prediction in patients with ESCC.

## 2. Patients and Methods

From July 2006 to December 2008, a retrospective analysis was conducted for patients with ESCC who underwent radical esophagectomy. The inclusion criteria were as follows: (1) ESCC confirmed by histopathology and classified by the 7th edition of TNM classification [10]; (2) curative esophagectomy (Ivor Lewis procedure or McKeown procedure) with standard lymphadenectomy (two-field or three-field lymphadenectomy) [11, 12]; (3) limited disease without distant metastasis; (4) without preoperative neoadjuvant therapy; (5) without previous anti-inflammatory medicines; and (6) preoperative laboratory results obtained before esophagectomy. At last, 277 patients were enrolled in the current study. Ethical approval was obtained from the Ethical Committees of Zhejiang Cancer Hospital (Hangzhou, China).

Patients were followed up with a clinical examination in our outpatient department every 3 to 6 months for the first 2 years after surgery and then annually. A cancer-specific survival (CSS) analysis was ascertained in the current study. The CSS was defined as the time from surgery to cancer-related death. The median follow-up was 42.6 months.

### 2.1. Statistical Analysis

Statistical analysis was conducted with SPSS 17.0 (SPSS Inc., Chicago, IL, USA) and R 3.1.2 software (Institute for Statistics and Mathematics, Vienna, Austria). Independent* t*-tests and chi-squared tests were used to compare clinical characteristics and RDW. The CSS was calculated by the Kaplan-Meier method. Univariate and multivariable analyses were used to examine the prognostic factors. A receiver operating characteristic (ROC) curve was plotted to verify the accuracy of RDW for CSS prediction. The area under curve (AUC) was used as an estimation of diagnostic accuracy. A nomogram for possible prognostic factors (including age and sex) associated with CSS was established by R software, and the predictive accuracy was evaluated by Harrell's concordance index (c-index) [13, 14]. A *P* value less than 0.05 was considered to be statistically significant.

## 3. Results

**Figure 1:**
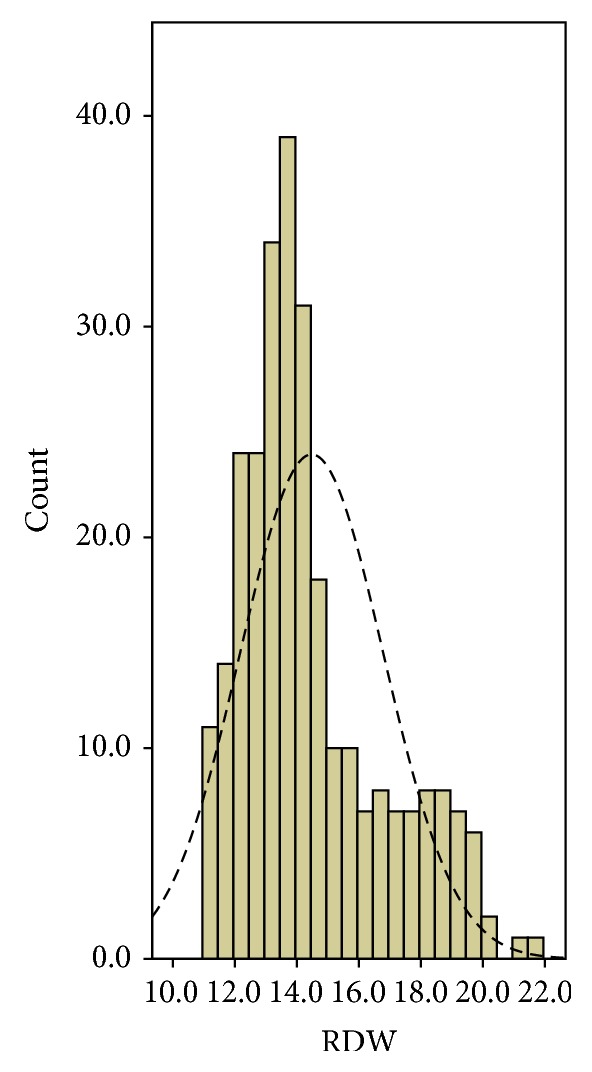
The histogram of the RDW.

Among the 277 patients, 37 (13.4%) were women and 240 (86.6%) were men. The histogram of RDW is shown in Figure 1. The mean value of RDW was 14.5 ± 2.3%. The upper normal range for RDW was 14.5%. Then patients were divided into two groups: RDW ≥14.5% and RDW <14.5%. Clinicopathologic characters between the high and low groups for RDW were shown in Table 1.

**Figure 2:**
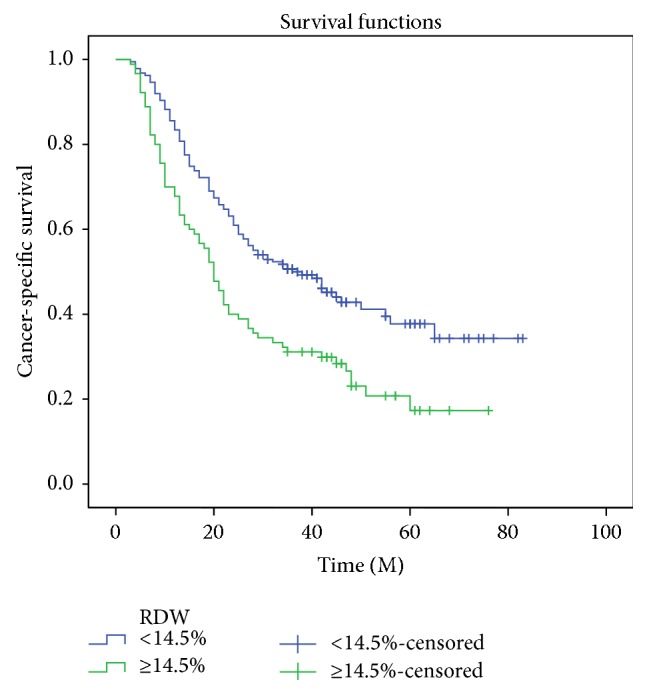
Kaplan-Meier CSS curves stratified by RDW. Patients with RDW <14.5% had a significantly better 5-year CSS than patients with RDW ≥14.5% (43.9% versus 23.3%, *P* < 0.001).

Patients with RDW <14.5% had a significantly better 5-year CSS than patients with RDW ≥14.5% (43.9% versus 23.3%, *P* < 0.001) (Figure 2). Then clinicopathological characters for CSS prediction were investigated by univariate and multivariate analyses. Our study demonstrated that RDW was an independent prognostic factor in patients with ESCC. RDW ≥14.5% had a hazard ratio (HR) of 1.396 (95% confidence interval (CI): 1.022–1.908, *P* = 0.036) for CSS (Table 2).

**Figure 3:**
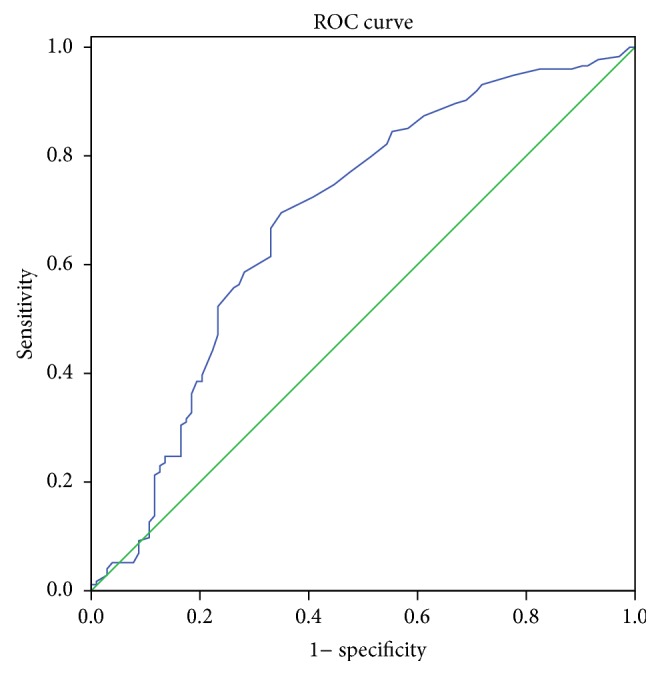
ROC curve for CSS prediction. A ROC curve plots the sensitivity on the *y*-axis against one minus the specificity on the *x*-axis. The area under curve (AUC) was used as an estimation of diagnostic accuracy. The AUC was 0.684 (95% CI: 0.616–0.752, *P* < 0.001). It demonstrated that RDW (cut-off point: 14.5%) predicts survival with a sensitivity of 44.3% and a specificity of 77.7%. Diagonal segments are produced by ties.

An ROC curve was also plotted to verify the accuracy of RDW for CSS prediction in the current study. The area under the curve (AUC) was 0.684 (95% CI: 0.616–0.752, *P* < 0.001). It demonstrated that RDW (cut-off point: 14.5%) predicts survival (CSS) with a sensitivity of 44.3% and a specificity of 77.7% (Figure 3).

**Figure 4:**
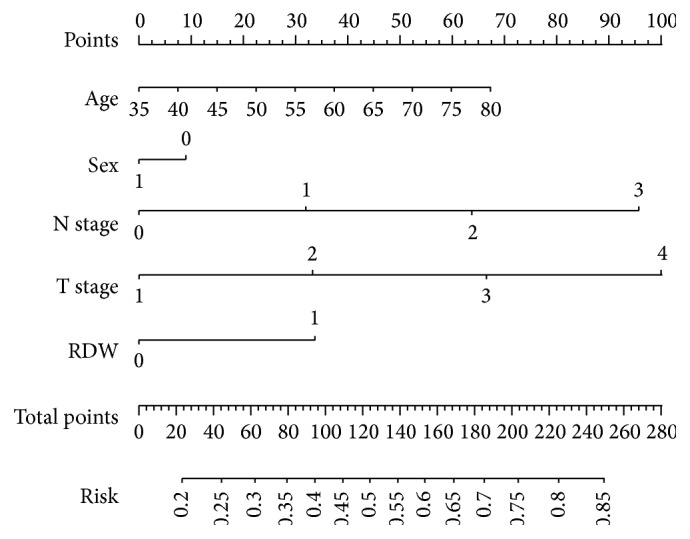
A nomogram predicts survival prediction based on RDW and other prognostic factors in patients with ESCC. The nomogram is used by totalling the points identified at the top of the scale for each independent factor. This total point score is then identified on the total points scale to determine the probability of risk prediction. Harrell's c-index for CSS prediction was 0.68. For example, a female (9 points) patient aged 60 years (38 points) with T2 (33 points), N1 (31 points), and RDW ≥14.5% (33 points) would score 144 total points which converts to a risk probability for death of 57%.

To predict the survival risk (CSS) for patients with ESCC, a novel model (nomogram) was established by prognostic factors combined with age and sex (Figure 4). It can predict the probability of death for patients with ESCC. Harrell's c-index for CSS prediction was 0.68.

## 4. Discussion

To the best of our knowledge, this is the first study to determine the prognostic value of RDW in predicting prognosis for patients with ESCC. We concluded two major findings: (1) RDW was associated with cancer stage; (2) RDW was associated with cancer prognosis. In addition, our study was also the first attempt to establish a predictive nomogram to improve predictive accuracy based on RDW.

RDW is a parameter in complete blood cell count. It has been reported as an inflammatory biomarker [7, 15]. The mechanism between inflammation and RDW has not been elucidated, but high levels of RDW are thought to be provoked by chronic inflammation and poor nutritional status [16, 17]. Recent studies demonstrated that RDW is associated with prognosis in several cancers, such as lung cancer [8] and prostate cancer [9]. Therefore, in the current study, we investigated the prognostic role of RDW in patients with ESCC. We concluded that patients with RDW <14.5% had a significantly better 5-year CSS than patients with RDW ≥14.5% (43.9% versus 23.3%, *P* < 0.001). Because the values of RDW were positively associated with cancer stage (T stage and N stage) in our study, it is not surprising that patients with higher RDW values had worse survival.

In the present study, we attempt to establish a predictive nomogram to predict the survival prediction based on RDW and other prognostic factors. We believe that our model could be a simple and easy tool for both the doctors and patients for estimating the survival in the absence of treatment in patients with ESCC. Thus, for example, a female (9 points) patient aged 60 years (38 points) with T2 (33 points), N1 (31 points), and RDW ≥14.5% (33 points) would score 144 total points which converts to a risk probability for death of 57%. Thus, we believe that nomogram based on CSS could be used as an accurately prognostic prediction for patients with ESCC.

Recent studies reported that SIR plays an important role in cancer progression [5, 6]. However, the prognostic value of inflammatory index in EC was not established. Thus, more and more inflammatory indexes were confirmed. In our previous study, we showed that lymphopenia is an independent predictive factor in patients with ESCC [18]. However, we also demonstrated in limitations that diabetes mellitus, renal and/or hepatic failure, and other inflammatory diseases may potentially affect the lymphocytes. In the current study, therefore, we attempted to investigate the new inflammatory index (RDW) in patients with ESCC. In addition, we created a nomogram based on the RDW and other associated risk factors. This can be an edge over using lymphocyte count model. We believe that our model could be a simple and easy tool for both the doctors and patients for estimating the survival in the absence of treatment in patients with ESCC. Based on the findings, we recommend that RDW, a very common, easy, and simple marker, should be considered as a potential prognostic biomarker in patients with ESCC.

Several limitations should be acknowledged in the current study. First, the current study was a retrospective study with a small sample. Second, the c-index showed that the model has a good accuracy but it is not perfect. Thus, there is still room for improvement of the predictive ability of the nomogram. The data in the current study was overlapped but from a different perspective. In our previous study, we mentioned the limitation that lymphopenia itself alone without other variables may not associate with the cancer prognosis. Thus, aim of current study was to investigate prognostic role of RDW. We concluded that RDW predicts survival similar to lymphocyte count and should be considered as an alternative to lymphocyte count. However, further studies are needed to illuminate the relationship between RDW and prognosis in patients with ESCC.

In summary, based on the findings of the current study, we concluded that RDW is a potential prognostic biomarkers in patients with ESCC. The nomogram based on CSS could be used as an accurately prognostic prediction for patients with ESCC.

## Figures and Tables

**Table 1:** Comparison of baseline clinical characteristics based on RDW.

	Cases (*n*)	RDW (%)	*P* value	RDW (%)		*P* value
		(mean ± SD)		<14.5 (*n*)	≥14.5 (*n*)	
Age (years)			0.759			0.931
≤60	158	14.5 ± 2.3		107	51	
>60	119	14.4 ± 2.3		80	39	
Gender			0.979			0.993
Female	37	14.5 ± 2.4		25	12	
Male	240	14.5 ± 2.3		162	78	
Tumor length (cm)			<0.001			0.003
≤3.0	78	13.6 ± 1.7		63	15	
>3.0	199	14.8 ± 2.4		124	75	
Tumor location			0.901			0.906
Upper/middle	143	14.5 ± 2.3		97	46	
Lower	134	14.5 ± 2.3		90	44	
Vessel invasion			0.732			0.895
Negative	232	14.5 ± 2.3		157	75	
Positive	45	14.4 ± 2.3		30	15	
Differentiation			0.401			0.493
Well/moderate	222	14.4 ± 2.2		152	70	
Poor	55	14.7 ± 2.6		35	20	
T stage			<0.001			<0.001
T1-2	97	13.6 ± 2.1		79	18	
T3-4	180	14.9 ± 2.3		108	72	
N stage			0.056			0.012
N0	150	14.2 ± 2.2		111	39	
N1–3	127	14.8 ± 2.3		76	51	

**Table 2:** Univariate and multivariate analyses of CSS in ESCC patients.

	CSS %	*P* value	Univariate analysis HR (95% CI)	*P* value	Multivariate analysis HR (95% CI)	*P* value
Age (years)		0.579		0.583	—	—
≤60	38.0		1.000			
>60	36.1		1.088 (0.806–1.468)			
Gender		0.182		0.189	—	—
Female	51.4		1.000			
Male	35.0		1.387 (0.851–2.260)			
Tumor length (cm)		<0.001		0.001		0.385
≤3.0	53.8		1.000		1.000	
>3.0	30.7		1.918 (1.328–2.769)		1.198 (0.797–1.799)	
Tumor location		0.331		0.337	—	—
Upper/middle	41.3		1.000			
Lower	32.8		1.157 (0.859–1.558)			
Vessel invasion		0.003		0.003		0.547
Negative	40.5		1.000		1.000	
Positive	20.0		1.738 (1.203–2.511)		1.125 (0.767–1.652)	
Differentiation		0.029		0.033		0.061
Well/moderate	39.2		1.000		1.000	
Poor	29.1		1.476 (1.033–2.109)		1.418 (0.984–2.043)	
T stage		<0.001		<0.001		0.011
T1-2	57.7		1.000		1.000	
T3-4	26.1		2.371 (1.668–3.369)		1.683 (1.129–2.508)	
N stage		<0.001		<0.001		<0.001
N0	54.7		1.000		1.000	
N1–3	16.5		2.846 (2.089–3.877)		2.279 (1.640–3.165)	
Adjuvant therapy		0.121		0.126	—	—
No	40.3		1.000			
Yes	30.2		1.277 (0.934–1.746)			
RDW (%)		<0.001		<0.001		0.036
<14.5	43.9		1.000		1.000	
≥14.5	23.3		1.719 (1.268–2.331)		1.396 (1.022–1.908)	
